# Total knee arthroplasty post-high tibial osteotomy, results of an early experience from a North African arthroplasty unit, and a comprehensive review of the literature

**DOI:** 10.1186/s13018-023-04199-1

**Published:** 2023-09-20

**Authors:** Ahmed M. Abdelaal, Ahmed A. Khalifa

**Affiliations:** 1 Orthopaedic Department, Assiut University Hospital, Assiut, Egypt; 2 Hospital for Advanced Orthopaedics, Assiut, Egypt; 3 Orthopedics Department, Qena Faculty of Medicine, South Valley University, Kilo 6 Qena-Safaga Highway, Qena, 83523 Egypt

**Keywords:** Total knee arthroplasty, High tibial osteotomy, Conversion, North African

## Abstract

**Purpose:**

To report an early experience after converting HTO to TKA by reporting the incidence of functional, radiological, and complications in a single surgeon case series from a North African specialized arthroplasty unit.

**Methods:**

Between 2010 and 2020, 33 knees in 31 patients (two bilateral) were operated upon, 24 females and seven males, had a mean age of 65 ± 4.5 years; 17 (51.5%) knees had medial wedge opening (WMO), while 16 (48.5%) had lateral wedge closure (LWC) osteotomies. The mean time from HTO to TKA was 8.1 ± 3.3 years. A posterior stabilized (PS) implant was used in 31 (93.9%), while in 2 (6.1%), a varus–valgus constrained (VVC) implant was used. A tibial stem was needed in 13 (39.4%) knees. The functional assessment was performed according to the Knee Society Scoring System (KSS). The radiographic assessment included the anatomical femorotibial angle (aFTA) for alignment, the medial proximal tibial angle (MPTA), and the tibial slope (TS).

**Results:**

After a mean follow-up of 4.3 ± 1.1 years, the KSS knee and function sub-scores improved from a preoperative mean of 41 ± 8.9 (26 to 57) and 37.7 ± 9.2 (25 to 55) points to 91.3 ± 3.8 (81 to 94) and 85.5 ± 5 (80 to 95) points at the last follow-up, respectively (*P* < 0.05). The preoperative knee flexion improved from a mean of 84.5° ± 15.9 (55 to 110) to 110.6° ± 9.3 (95 to 125) (*P* < 0.05). The aFTA improved from a preoperative mean of 182.2° ± 10.3 (164 to 205) to a postoperative mean of 186° ± 2.6 (179 to 190) (*P* < 0.05). The MPTA changed from a preoperative mean of 88.4° ± 6.7 (77 to 102) to a postoperative (tibial component alignment) mean of 90° ± 1.7 (85 to 94) (*P* < 0.05). The mean preoperative TS changed from 80.9° ± 7.3 (68 to 96) to a mean postoperative of 86.9° ± 1.3 (83 to 89) (*P* < 0.05). Non-progressive radiolucent lines were detected at the tibial component in four (12%) knees. Complications were reported in seven (21.2%) knees; no revision was needed in any knee.

**Conclusions:**

The authors’ early experience showed improved functional and radiological outcomes; however, the complication incidence was relatively high, but no knees required revision. A longer follow-up is mandatory to prove the consistency of the results.

## Introduction

High tibial osteotomy (HTO) is still considered a valid and effective option for managing knee osteoarthritis with varus deformity, especially in relatively younger patients; it could be performed through a medial wedge opening (MWOHTO), lateral wedge closure (LWCHTO), or a dome osteotomy [1–4]. However, the survival rate of such a procedure decreases between 95 and 84% at five years and 79% and 65% at ten years, with eventual conversion to total knee arthroplasty (TKA) [5–10].

TKA post-HTO carries some challenges and is considered more complex than a primary TKA; several factors could increase the difficulty of the surgery, including but not limited to the presence of previous skin incisions, the hardware used for fixation after HTO, changes in the proximal tibial morphology, status of the soft tissue envelop, and patellofemoral joint alterations [8, 11–16]. Based on the previously mentioned factors, the surgeon will determine the type of surgical approach, TKA prosthesis, and if supplements such as tibial stems or bone defect reconstruction tools will be needed [13, 15, 17–19].

Despite the technical challenges related to TKA post-HTO, various reports from different groups, including registry-based studies, showed comparable results related to functional and radiological outcomes and survival rates in cases with TKA post-HTO vs. primary TKAs [13, 19, 20]. Furthermore, functional and radiological outcomes after converting MWOHTO or LWCHTO showed similar results [21, 22].

Plenty of reports in the literature discuss converting HTO to TKA [14, 19, 23], although HTO is commonly practiced in our area [24, 25]; however, to the best of our knowledge, no reports on converting HTO to TKA were published by surgeons practicing in our area. This study aimed to report an early experience after converting HTO to TKA by reporting the clinical, radiological, and complication incidence in a single surgeon case series from a North African specialized arthroplasty unit. Furthermore, after comprehensively reviewing the literature, a comparison was made between the current study results and previous reports.

## Patients and methods

The current study was a single surgeon retrospective case series including all patients who had TKA post-HTO operated upon by a surgeon with over 15 years of experience. Where in the period between 2010 and 2020, 38 patients (41 knees) were operated upon; seven patients were lost during follow-up, leaving 31 patients (33 knees) to be included in the current study (two bilateral TKA) having a mean follow-up of 4.3 (2 to 7) years. The patients’ mean age at the time of TKA was 64.6 (57 to 73) years; 24 (77.4%) were women, and the mean BMI was 28 (19 to 35).

### Patients’ evaluation

Patients were evaluated preoperatively and postoperatively (every year after surgery), and the data collected during the last follow-up were compared to the preoperative values. Functional assessment was performed according to the knee society score (knee score (KS) and the functional score (FS) sub-scores) [26], while the range of motion (ROM) was evaluated using a manual goniometer. Radiological assessment was performed on a weight-bearing short anteroposterior (AP) and lateral knee radiographs, and the following measurements were collected [27, 28]: 1—coronal alignment measured as the anatomical femorotibial angle (FTA), where a neutral anatomical coronal alignment was considered as 187° ± 3 (values below are varus and above are valgus), 2—The medial proximal tibial angle (MPTA), which postoperatively represented the tibial component coronal plane alignment, 3—tibial slope (TS), and 4—Insall–Salvati ratio (ISR). Furthermore, radiolucent lines developed at any time during follow-up were assessed [27].

### Operative details

#### Previous HTO characteristics

Seventeen (51.5%) knees had MWOHTO, while LWCHTO was performed in 16 (48.5%) knees. A T-plate was used for HTO fixation in 20 (60.7%) knees (15 were usual non-locking and five were locked plates), stables were used in eight (24.2%) knees, and a wedge plate was used in five (15.2%) knees. The mean time to convert HTO to TKA was 8.1 ± 3.3 (3 to 15) years. The mean preoperative anatomical FTA was 182° ± 10.3 (164 to 205), where neutral coronal knee alignment was defined in nine (27.3%) knees, 17 (51.5%) were in varus, and seven (21.2%) were in valgus. In three (9.1%) knees, the TS was reversed.

#### TKA surgical procedure details

All patients were operated upon under spinal anesthesia and tourniquet control. All surgeries were performed through a medial parapatellar approach; however, the skin incision was incorporated with the previous HTO incision in 20 (60.6%) knees, while a separate incision with an optimum skin bridge was utilized in 13 (39.4%) knees (all of them were in LWCHTO knees). After performing an initial exposure and before attempting patella eversion of mobilization, a pin was used to secure the patellar tendon. Extensile approaches (rectus snip or tibial tubercle osteotomy (TTO)) were not performed in any knee. In 30 (90.9%) knees, the hardware fixing the HTO was removed during the same TKA setting. By comparison, in two (6.1%) knees, the hardware was removed in another session before TKA; in one (3%) knee (MWOHTO), the plate was retained in situ, and only the proximal screws were removed, in another knee (3%), the heads of the proximal screws were broken, so the screw shafts were retained. In two (6.1%) knees, there was evidence of fibrous nonunion; both were MWOHTO. The TKA was performed adopting the measured resection philosophy aiming at achieving neutral mechanical alignment, and ligament balancing was performed, as necessary.

**Fig. 1:**
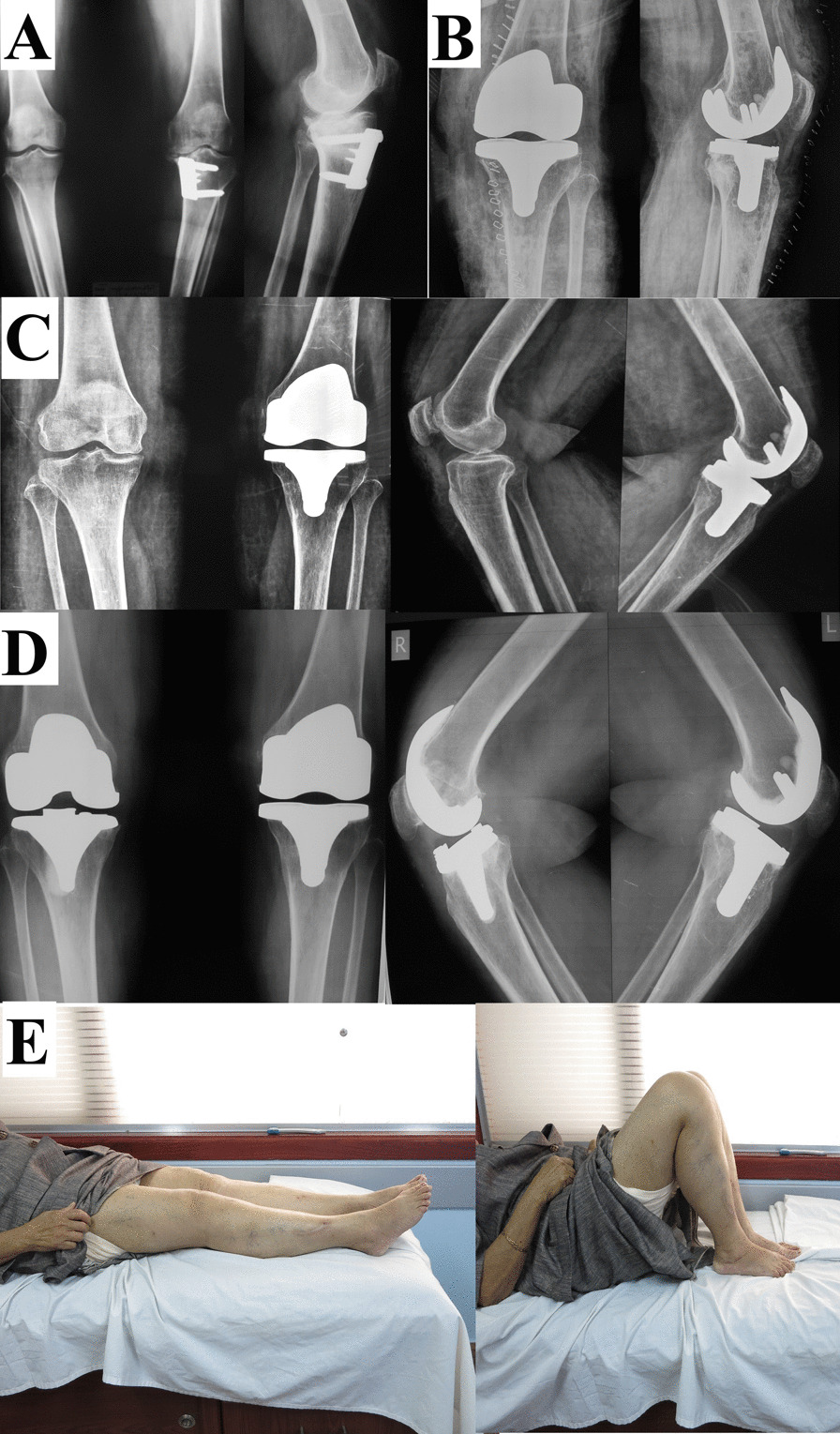
Female patient, 68 years old. **A** MWOHTO performed in 2006 using a medial wedge plate. **B** immediate postoperative after conversion to TKA using a PS implant in January 2013. **C** Follow-up in August 2015. **D** Last follow-up (after having a contralateral TKA) in August 2018 (five years follow-up), showing proper implant positioning with no loosening. **E** Clinical images at the last follow-up showed full knee flexion and extension

**Fig. 2:**
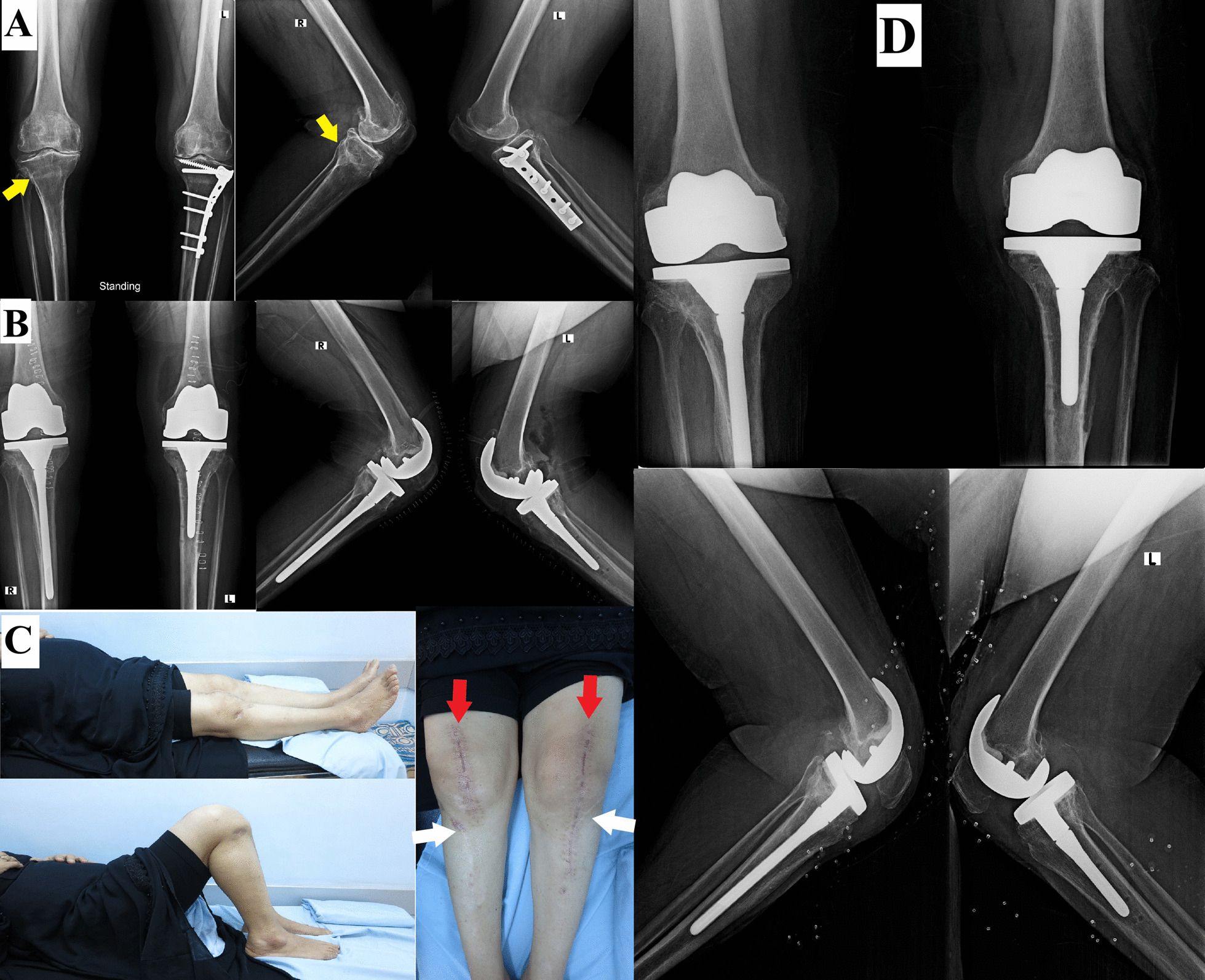
Female patient, 62 years old. **A** Bilateral LWCHTO (right in 2003 and left in 2004) using a non-locked T-plate (the plate on the right side was removed in a prior session, and the united HTO is shown (yellow arrowhead)). **B** Bilateral TKA was performed at the same session in 2014, using a PS implant and tibial stem bilaterally. **C** At six months follow-up, the clinical images showed optimum knee flexion and extension and good healing of the skin incision (red arrowheads showing TKA skin incision, white arrowheads showing the previous HTO skin incision). **D** At five years follow-up, both knees showed proper implant positioning and no loosening

**Fig. 3:**
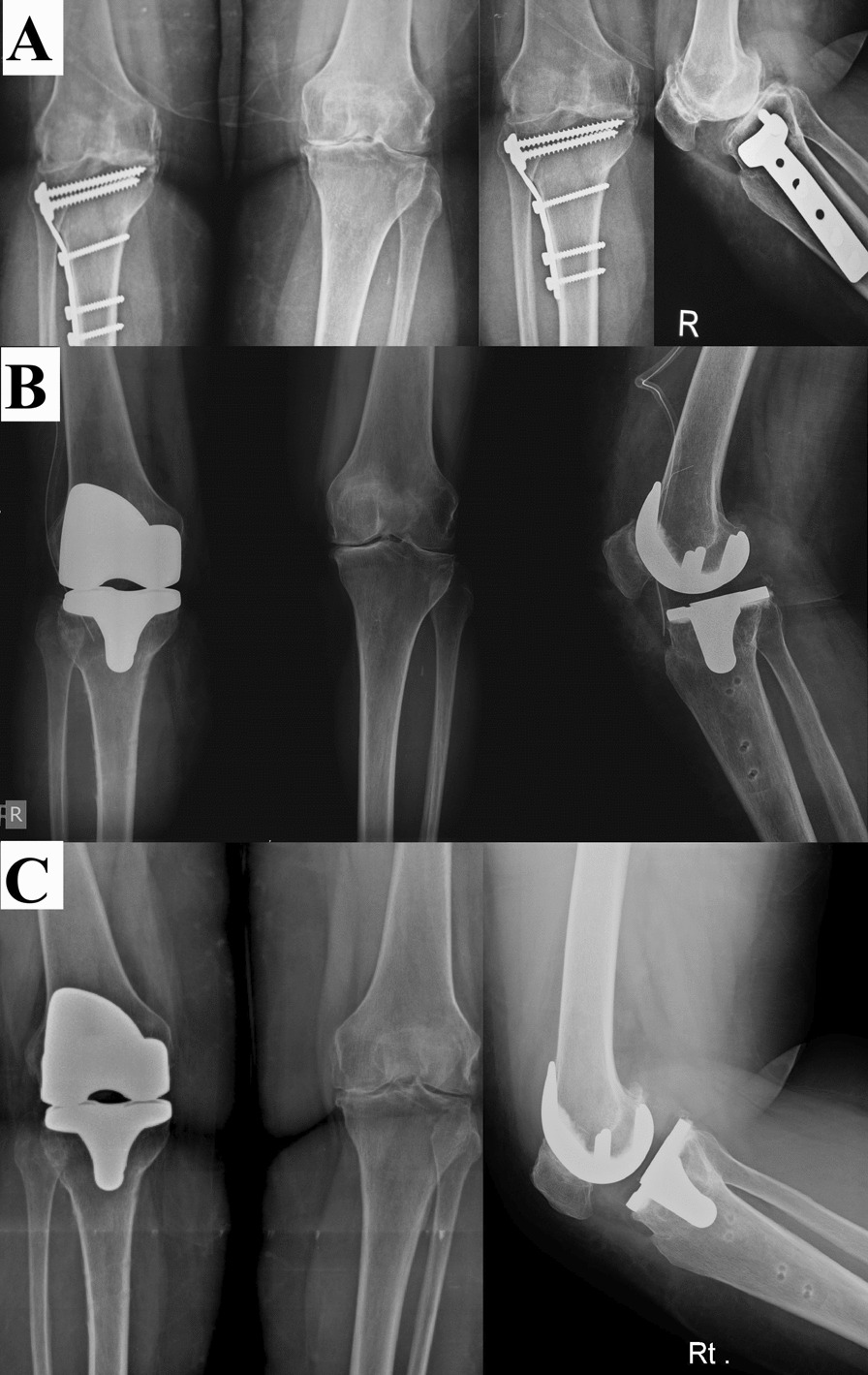
Female patient, 65 years old. **A** LWCHTO performed in 2005 using a long non-locked T-plate. **B** Immediate postoperative after conversion to TKA using a PS implant in May 2016. **C** Last follow-up in September 2020, showing proper implant positioning with no loosening

**Fig. 4:**
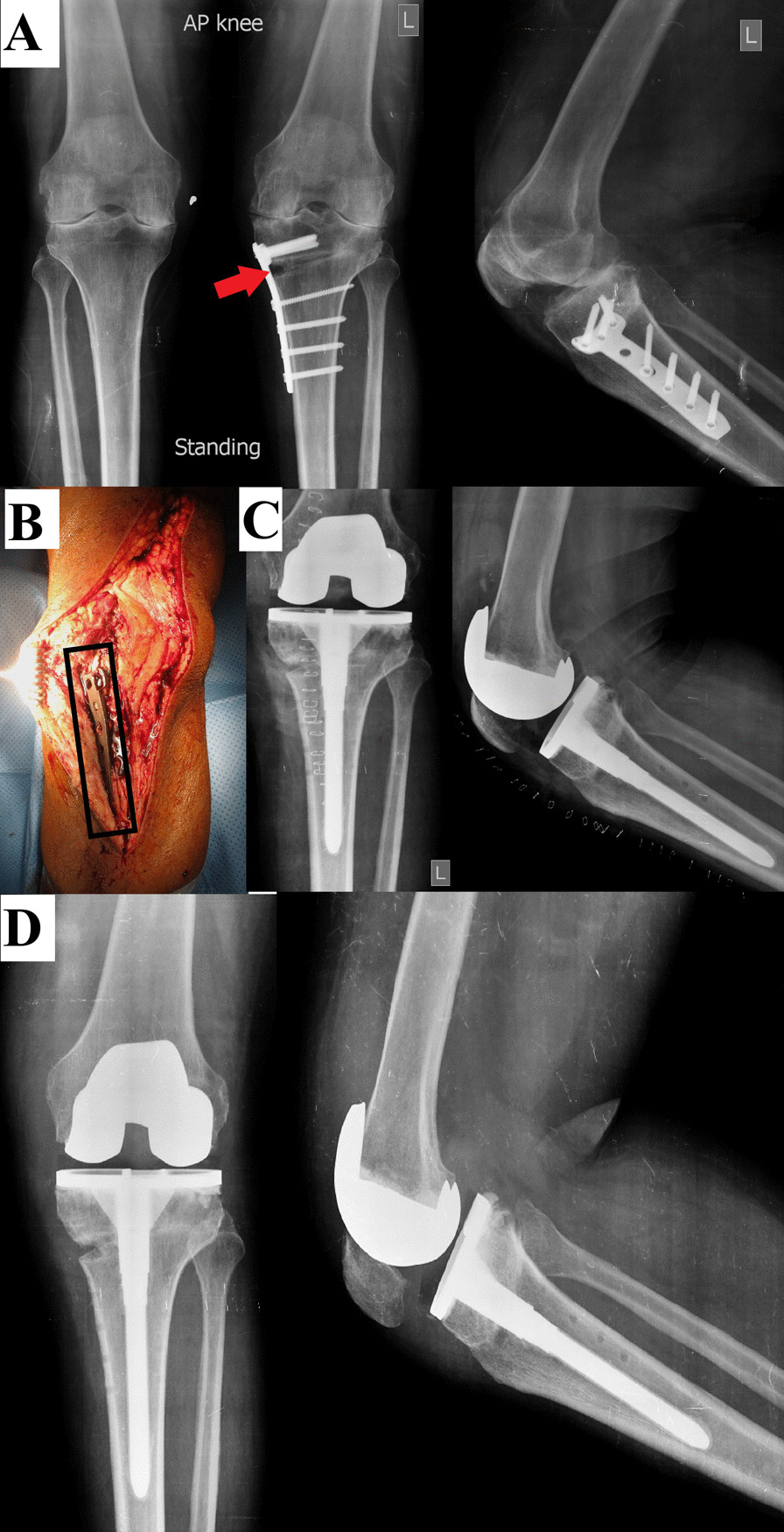
Male patient, 59 years old. **A** MWOHTO performed in 2014 using a locked T-plate, preoperative radiographs showed signs of nonunion (red arrowhead). **B** Intraoperative images while the knee was approached through a medial parapatellar approach, showing the extent of the medial plate. **C** Immediate postoperative after conversion to TKA using a PS implant and a tibial stem in February 2017. **D** After three years of follow-up, although the medial HTO gap did not fully unite, the implants showed preserved proper positioning

Regarding TKA implants used, in all patients, a cemented prosthesis was used. [A posterior stabilized (PS) implant was used in 31 (93.9%), where four (12.1%) of them had a rotating platform (RP), while a varus–valgus constrained (VVC) implant was used in two (6.1%) knees.] A tibial stem was needed in 13 (39.4%) knees; one was an offset stem. A medial tibial augment was used in one (3%) knee to reconstruct a medial tibial defect. The patella was not resurfaced in any knee (Figs. 1, 2, 3, 4).

Basic patients’ demographics and surgical characteristics are shown in Table 1.

**Table 1:** Basic demographic and surgical characteristics of the included patients

Parameters		Values
*Patients*		
No. of patients		31
No. of knees		33 (two bilateral)
Gender^a^	Male	7 (22.6)
	Female	24 (77.4)
Age		64.6 ± 4.5 (57 to 73)
Side^a^	Right	19 (57.6)
	Left	14 (42.4)
BMI^b^		28 ± 4.1 (19 to 35)
Follow-up (years)^b^		4.3 ± 1.1 (2 to 7)
*HTO surgical characteristics*		
Time from HTO to TKA (years)^b^		8.1 ± 3.3(3 to 15)
Osteotomy type^a^	MWOHTO	17 (51.5)
	LWCHTO	16 (48.5)
Fixation device^a^	Non-locked T-PLATE	15 (45.5)
	Staples	8 (24.2)
	Wedge plate	5 (15.2)
	locked T-PLATE	5 (15.2)
*TKA surgical characteristics*		
Skin incision^a^	Same as HTO	20 (60.6)
	Separate incision	13 (39.4)
HTO hardware removal^a^	In the same setting	30 (90.9)
	In a prior session	2 (6.1)
	retained	1 (3)
Knee prosthesis^a^	PS^c^ (non-constrained)	31 (93.9)
	VVC	2 (6.1)
Extras^a^	Tibial stem^d^	13 (39.4)
	Augment	1 (3)

*BMI* body mass index, *HTO* high tibial osteotomy, *MWOHTO* medial wedge opening high tibial osteotomy, *LWCHTO* lateral wedge closure high tibial osteotomy, *TKA* total knee arthroplasty, *PS* posterior stabilized, *VVC* varus–valgus constrained

^a^The values are given as the number of patients, with the percentage in parentheses

^b^The values are given as the mean ± standard deviation, with the range in parentheses

^c^In four knees, a rotating platform was used

^d^One was an offset stem

### Ethical statement and statistical analysis

This study was conducted per the ethical standards of the Helsinki Declaration. No experimental maneuvers were performed on any patient; the ethical committee of our institution waived the approval as this was considered usual patient care. Statistical analysis was performed using SPSS Statistics, version 25 (IBM Corp., Armonk, New York). Qualitative variables were expressed as numbers and percentages. The continuous variables were expressed as a mean ± standard deviation (range). Student’s t tests were used to compare the mean functional and radiological scores between pre- and postoperative values, with p-values of less than 0.05 considered statistically significant.

## Results

### Functional outcomes

The knee society knee score (KSKS) and the knee society functional score (KSFS) improved significantly from a preoperative mean of 41 ± 8.9 (26 to 57) and 37.7 ± 9.2 (25 to 55) points to 91.3 ± 3.8 (81 to 94) and 85.5 ± 5 (80 to 95) points at the last follow-up, respectively (*P* < 0.05). The preoperative knee flexion improved from a mean of 84.5° ± 15.9 (55 to 110) to 110.6° ± 9.3 (95 to 125) (*P* < 0.05).

### Radiological outcomes

The anatomical FTA improved from a preoperative mean of 182.2° ± 10.3 (164 to 205) to a postoperative mean of 186° ± 2.6 (179 to 190) (*P* < 0.05), where 27 (81.8%) knees were neutrally aligned, while four (12.1%) and two (6.1%) knees were in varus and valgus alignment, respectively. The MPTA changed from a preoperative mean of 88.4° ± 6.7 (77 to 102) to a postoperative (tibial component alignment in the coronal plane) mean of 90° ± 1.7 (85 to 94) (*P* < 0.05). The mean preoperative TS changed from 80.9° ± 7.3 (68 to 96) to a mean postoperative of 86.9° ± 1.3 (83 to 89) (*P* < 0.05). The ISR changed from a mean preoperative of 1.2 ± 0.3 (0.5 to 1.6) to a postoperative mean of 1.3 ± 0.2 (0.8 to 1.7) (*P* = 0.14). Radiolucent lines were detected at the tibial component bone interface in four (12.%) knees, at zones 1 and 2 in two knees, zone 4 in one knee, and zone 6 (AP), three (lateral) in one knee according to the Knee Society Roentgenographic Evaluation and Scoring System [29]; however, these were non-progressive.

### Complications

Complications were reported in seven (21.2%) knees, three (9.1%) knees required manipulation under anesthesia for stiffness, two (6.1%) knees had superficial wound infection which was treated conservatively and resolved after daily dressing, one (3%) knee had a common peroneal nerve palsy postoperatively which resolved after three months spontaneously, and one (3%) knee had an intraoperative patellar tendon avulsion which was managed by direct repair and fixation using a stable, the patient had an extension lag of 10° postoperatively. No knees required revision till the last follow-up.

## Discussion

Although the current case series was limited by the relatively small number of patients and a short follow-up, however, the authors’ early experience performing TKA post-HTO resulted in functional and radiological outcomes improvement with limited use of higher constrained implants. Furthermore, a relatively high complication rate was encountered; however, no patient required revision till the last follow-up.

In the literature, there has been controversy regarding the outcomes and survival of TKA post-HTO compared to primary TKA [11, 13, 23, 30, 31], which proves the variability, technical difficulties, and challenges that could be faced with TKA post-HTO cases. In a systematic review and meta-analysis by Sun et al.[19] comparing the results of TKA post-HTO to primary TKA, they included 16 studies (3955 patients post-HTO and 99,597 patients primary TKA), and the authors found no difference regarding functional outcomes (KSS), radiological measures, or survival rates (at ten years follow-up). Similar survival rates as primary TKA were also confirmed in a study by Niinimäki et al. [13] and Badawy et al. [30], where the survival reached 92% and 88% at 10 and 15 years, respectively. On the contrary, in a study based on the Danish Knee Arthroplasty Registry, the authors reported 91% survival for TKAs post-HTO compared to 94% for primary TKAs; it is worth mentioning that the survival difference did not persist after controlling for the younger age and predominant male sex for the TKA post-HTO cohort [11].

- The time between HTO and conversion to TKA:

The time to convert HTO to TKA varied among studies and after various HTO techniques. In the current series, HTO was converted to TKA after a mean of eight years. In a study by Hevesi et al. [32] evaluating 70 patients (140 knees) who had a primary TKA in one knee and a TKA post-HTO in the other knee (94% LWCHTO), HTO conversion was performed after a mean of 14 ± 7 years. In a study by Ehlinger et al. [12] comparing the results of TKAs performed after either MWOHTO (58 knees) vs. LWCHTO (77 knees), the mean time from HTO to TKA was 11.2 years, they reported that it was significantly longer in knees which had an LWCHTO (*P* < 0.001).

- Surgical approaches utilized during converting HTO to TKA:

One of the challenges is the presence of previous skin incisions, which differ in position and length according to the type of osteotomy and the implant used for fixation [33, 34]. The surgeon should determine beforehand if the same incision will be incorporated in the TKA surgical approach or if a different incision will be used; if the latter is the case, at least 6 cm of skin bridge should be considered for optimum healing [34, 35]. All knees in the current series were operated through a medial parapatellar approach, incorporating the same HTO incision in 60.6% of the knees; however, in 39.4%, a separate skin incision was required, as the original incision was located laterally for LWCHTO. In the Ehlinger et al. study [12], the authors reported incorporating the original HTO incision in only 54.2% of the cases; furthermore, they found that two incisions were required more in knees that had a previous LWCHTO.

Another issue is the need to perform extensile approaches, as difficulty while mobilizing or everting the patella could be encountered [34, 36]. To avoid the undue risk of patellar tendon avulsion, the surgeon might consider using a more extensile approach, like performing TTO or a rectus snip [35]. Hevesi et al. [32] reported that they tend to use the safest approach possible, considering whether hardware removal is needed at the same setting and whether this will be achieved through the same approach for TKA or not. No extensile approaches (TTO or rectus snip) were needed in their series. We reported the same results in the current study, as no extensile approach was needed in any knee. In contrast, in a study by Filho et al. [21], there was a trend toward using additional exposure techniques in LWCHTO knees (TTO in 22.2% and quadriceps snip in 2.6%).

- Removing HTO fixation hardware:

In the study by Hevesi et al. [32], in 63% of the knees, no hardware removal was performed; removal at the same setting of TKA surgery in 34% of the knees (half of them had partial removal of the hardware, either isolated screws removal or even only part of the screws was removed); in 3%, the hardware was removed as part of a staged procedure to prepare for TKA. This was contrary to the current series, whereas in 90.9% of the knees, the hardware was removed during the same TKA setting, and in only one knee, the plate was retained in situ (however, the proximal screws were removed) as this was a long T-plate used to fix MWOHTO, and by removing the plate, it could have led to medial soft tissue structures stripping. However, we had the same trend as Ehlinger et al. [12], where hardware removal was performed in most patients (98.5%); however, this was performed as a single stage in only 47.3% of the knees and was more common in knees with MWOHTO.

- Type of TKA prosthesis, constrained level, and the need for extras:

Previous studies showed acceptable outcomes and durable survival rates when using non-constrained implants (mainly PS and CR prosthesis); however, based on the complexity of the surgery, soft tissue status, and the need for extensive soft tissue releases, the surgeon could use a higher level of constraint (VVC or even a Hinged knee) [11, 13, 18].

In the current study, a cemented prosthesis was used in all knees, where a PS implant was used in most of the knees (93.3%), while a VVC implant was needed in only two (6.1%) knees. In a study based on the Finnish Arthroplasty Register, Niinimäki et al. [13] evaluated 1063 TKAs post-HTO; non-constrained implant was used in 96.5% (78.8% CR vs. 17.7% PS), while in 3.6% of the knees, a higher constraint was used (3% VVC and 0.6% hinged). In a study by Chalmers et al. [15] retrospectively reviewing their results of 231 TKA post-HTO over 12 years, the mean age of the patients was 64 years, and most of them (87%) had a prior LWCHTO. A cemented PS implant was used in 93%, while 4% required a VVC prosthesis. In Hevesi et al. [32] series, in most of the patients (97%), a non-constraint prosthesis was used (96% PS vs. 1% CR), while a VVC prosthesis was used in 3%; however, the authors reported that implants characteristics were not different from those used for primary TKA. In the study by Ehlinger et al. [12], a PS implant without further constraint was used in all knees. Furthermore, no significant differences were found between fixed and mobile bearing knees, as in a study by Hernigou et al. [37] compared the results of using either a fixed (57 knees) or mobile bearing (41 knees) prosthesis while performing TKA post-HTO (all were MWOHTO). After a mean follow-up of 17 years, the authors reported no difference regarding the functional outcomes according to the KSS between both groups, but the ROM was slightly better in the fixed bearing group (117° vs. 110°).

- To add a tibial stem or not:

Using a tibial stem varied among the previous studies (reaching up to 13%), the decision to use or not to use a stem is according to the surgeon’s preference [12, 15, 32, 35, 38]. In the current series, tibial stem was utilized in 39.4% of the knees (one offset), which is considerably higher than what was reported in the literature; we preserved using tibial stems for patients with higher BMI when a long medial plate was used for fixing MWOHTO to avoid stress risers at the site of distal screw holes, and in cases, if the osteotomy showed signs of nonunion (which we had in two knees). Song et al.[8] and Yoshino et al.[39] reported treating the osteotomy site nonunion by adding a tibial stem after curetting the fibrous tissue and using an autogenous bone graft. Kuwashima et al. [40] alluded to the possibility of proximal tibial anatomical changes, which could affect the implant positioning or necessitate using offset stems to avoid implant impending on the cortices; furthermore, the possible changes in the anatomical or mechanical axes could hinder the smooth introduction of a longer tibial stem.

- Radiographic outcomes:

Preoperatively, knees with previous HTO showed a wide range of coronal plane malalignment, either recurrence of varus deformity or overcorrections. As in a study by Carnero et al. [41] on the TKA post-HTO long-term results (all were LWCHTO), the authors included 41 TKAs performed post-HTO and followed up for a minimum of ten years and compared them with a matched group of 41 primary TKAs, and the mean preoperative FTA in patients with previous HTO was 4° of varus (ranging between 6° of valgus and 7.1° of varus). This variability was also noted in the current study, where the mean preoperative FTA was 182° ± 10.3 (5° of varus), ranging from 164° to 205° (23° varus to 18° valgus). In a study by Treuter et al. [16] evaluating the long-term outcomes for TKA performed post-HTO (closing wedge according to Wagner), they included 48 patients followed up for a mean of 13.3 years, and the authors reported a preoperative mean FTA of varus 0.8° (ranging from varus 14° to valgus 8°). Even with the wide range of preoperative deformity, postoperatively, we achieved significant coronal plane alignment correction to a mean postoperative FTA of 186° ± 2.6 (1° of varus) (*P* < 0.05); furthermore, the range of alignment decreased to be between 179 and 190 (8° varus and 3° valgus). Moreover, in the Carnero et al. study [41], the alignment was improved postoperatively to a narrower range of 6° to 7° of valgus. Treuter et al. [16] reported that the FTA was corrected postoperatively to a mean of valgus 7.6° (valgus 2–9°).

In the current study, there was an improvement in the MPTA from a preoperative mean of 88.4° ± 6.7 (77 to 102) to a postoperative mean of 90° ± 1.7 (85 to 94) (*P* < 0.05); furthermore, the mean preoperative TS changed from 80.9° ± 7.3 (68 to 96) to a mean postoperative of 86.9° ± 1.3 (83 to 89) (*P* < 0.05); these indicate the proper alignment of the tibial component. The previously mentioned results were in accordance with Treuter et al. [16], who reported an MPTA of 90.0° (85 to 96) and a mean tibial slope of 88° (ranging from − 3 to 8).

In cases of TKA post-HTO, there is a concern about an increased incidence of tibial component loosening [42]; however, various studies reported that the presence of radiolucent lines was not indicative of increasing revision rates [16, 37, 41]. The incidence of tibial component-related radiolucent lines in the current series was 12.1%; however, these were non-progressive. In a study by Carnero et al. [41], tibial radiolucent lines were detected in six knees (14.6%); however, the authors reported that these were non-progressive. In a study by Hernigou et al. [37], radiolucencies around the tibial implant were present in six knees (4 FB and 2 MB, *P* = 0.24); however, this was < 2 mm and non-progressive. In a study by Treuter et al. [16], although the authors reported radiolucency related to the tibial component (13 knees in the AP and 11 in the lateral view), they were non-progressive. They had no revision for aseptic loosening required in any patient. Even with a cementless implant, as in a study by Batailler et al. [38], comparing cementless TKA prostheses post-HTO (41 knees) with a matched group of primary cementless TKA (82 knees). After a mean follow-up of 7.8 ± 2.4 years, the authors reported no radiolucent lines related to the tibial component.

- Functional outcomes:

Although acceptable functional outcomes and ROM in TKA post-HTO were achieved, these were variable among different studies compared to primary TKA [23, 38, 41, 42]. In the current study, no comparison with a primary TKA group was performed; however, all patients achieved considerably improved functional outcomes as the KSKS and KSFS changed significantly at the last follow-up to a mean of 91.3 ± 3.8 and 85.5 ± 5 points; furthermore, the knee flexion improved to a mean of 110.6° ± 9.3. Hevesi et al. [32] reported significant improvement in the KSKS (from 41 ± 16 to 82 ± 9) and KSFS (from 59 ± 16 to 82 ± 21) (*P* < 0.001), and the improvement in the functional outcomes was comparable to the primary TKA group at 5, 10, and 15 years of follow-up. In the study by Chalmers et al. [15], after a mean follow-up of eight years, the KSS improved significantly from a mean preoperative score of 59 points to a mean postoperative score of 93 points, as well as the knee flexion range which improved from 104 to 112 (*P* < 0.001). In a study by Treuter et al. [16], the functional score improved significantly according to the overall KSS from 93.2 points preoperatively to 160.8 points postoperatively (knee score from 63.1 to 91.3 and the function score from 35.6 to 90).

Furthermore, in the current series, comparing the outcomes between knees with MWOHTO and LWCHTO was not carried out; however, in the study by Filho et al. [21], all knees showed significant improvement in the functional and radiological outcomes compared to the preoperative scores, with no difference between both techniques. The same previous results were confirmed in a systematic review by Han et al. [22], where they reported comparable functional and radiological results in TKAs after either LWCHTO or MWOHTO.

- Complications and Revisions:

In the current study, a relatively high overall complications incidence (21.1%) from seven knees was encountered; most of the complications were due to stiffness (three) and superficial infection (two), but all were treated conservatively, and no revision was required in any patients till the last follow-up. Chalmers et al. [15] reported a 6% incidence of perioperative complications (excluding revisions or reoperations), and 4% of the knees had arthrofibrosis and required manipulation under anesthesia for stiffness; however, this was not affected by the type of the previous osteotomy (MWO vs. LWC); furthermore, a revision was required for 20 (9%) knees, 7% were aseptic revisions, and 2% were for septic failure. In the study by Ehlinger et al. [12], the early complications were more in the MWOHTO group (12.3% vs. 8.3%), but the difference was insignificant. However, late complications were significantly more in the LWCHTO group (12% vs. 6%). In a systematic review and meta-analysis of 15 studies by Seo et al. [23] comparing outcomes after TKAs post-HTO (3563 patients) compared to primary TKAs (71,281 patients), complication incidence was not significantly different between groups. However, on the contrary, in a more recent systematic review and meta-analysis by Chen et al. [14], they included 144,692 patients (4646 post-HTO vs. 140,074 primary TKAs) from 14 studies and reported that the complications and revision rates were higher in TKA post-HTO.

**Fig. 5:**
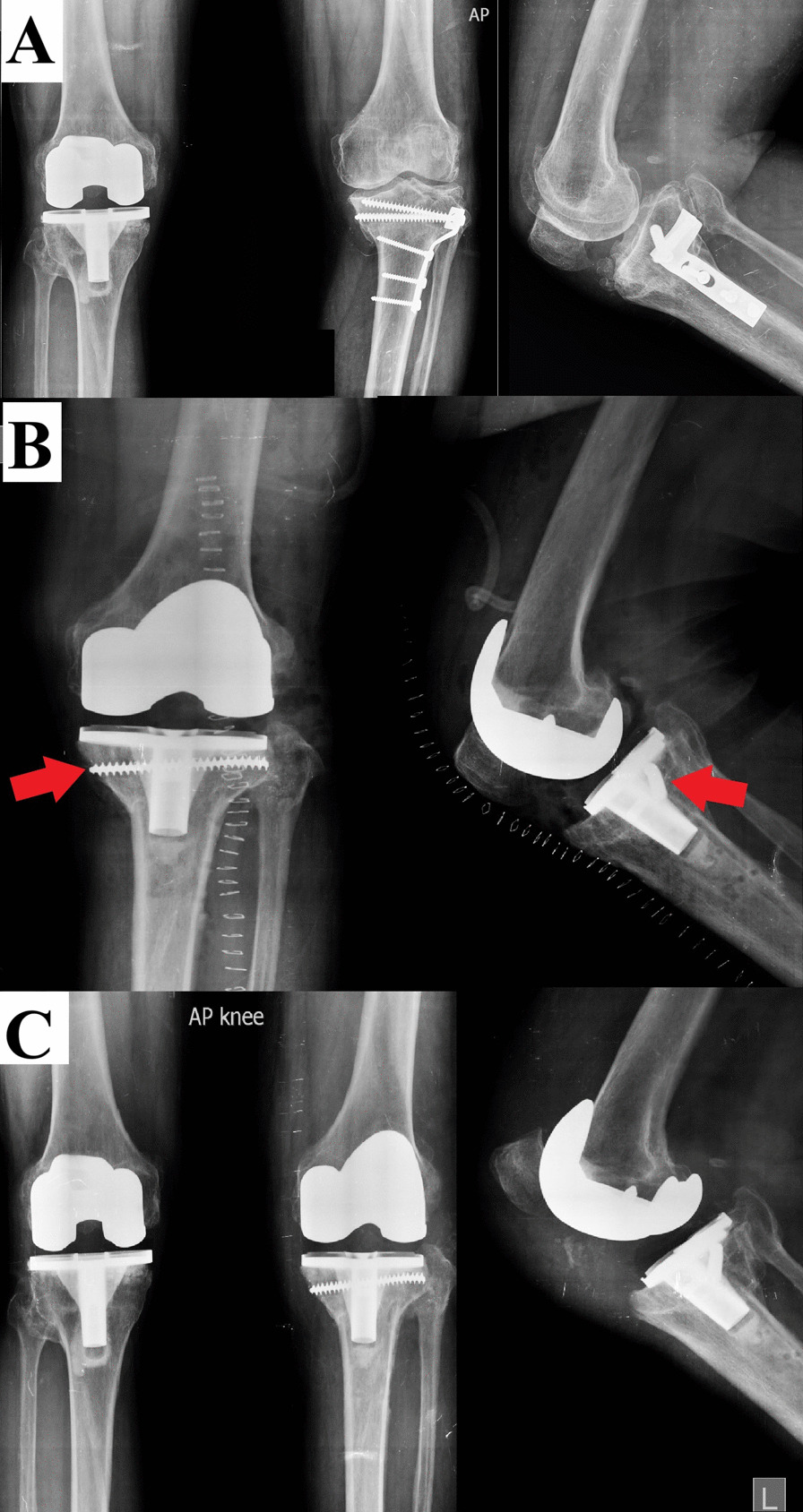
Female patient, 61 years old. **A** LWOHTO performed in 2004 using a non-locked T-plate. **B** Immediate postoperative after conversion to TKA in March 2015, broken proximal screws were retained, which did not interfere with the tibial component keel. **C** Last follow-up in August 2020, showing proper implant positioning with no loosening

Finally, the current study has some limitations. First, being a single-center retrospective study makes it prone to selection bias and lack of generalizability. Second, the small number of the included patients, which was further affected by lost patients during follow-up, hindered us from comparing various variables. Third, although we reported an early experience, a longer follow-up is still needed. Fourth, using short films for assessing the radiological parameters. Fifth, the tibial radiolucent lines were assessed in plain radiographs, and no further assessment using a CT scan was performed. Finally, comparing the results with a matched group of patients with primary TKA was not performed (Fig. 5).

## Conclusions

This series represented an early experience from a North African arthroplasty unit on a group of patients who had TKA post-HTO, which resulted in acceptable functional, radiological, and complications outcomes at a short-term follow-up. The authors were able to use non-constrained implants in most of the patients; however, a tibial stem was needed in a considerable number of knees. Although there were no revisions, a longer follow-up is essential to prove the results’ consistency, the implants’ survival, and to detect any late complications. Comparing the results with a matched group of primary TKA is still required.

## Data Availability

All the data related to the study are mentioned in the manuscript; however, the raw data are available with the corresponding author and will be provided on a written request.

## Abbreviations

HTO: High tibial osteotomy
MWOHTO: Medial wedge opening
LWCHTO: Lateral wedge closure
TKA: Total knee arthroplasty
ROM: Range of motion
AP: Anteroposterior
FTA: Anatomical femorotibial angle
MPTA: Medial proximal tibial angle
TS: Tibial slope
ISR: Insall–Salvati ratio
TTO: Tibial tubercle osteotomy
PS: Posterior stabilized
RP: Rotating platform
VVC: Varus–valgus constrained
KSKS: The knee society knee score
KSFS: Knee society functional score
